# Meniscal Substitution With a Semitendinosus Tendon Autograft

**DOI:** 10.1002/atn2.70088

**Published:** 2026-05-12

**Authors:** Riccardo Cristiani, Karl Eriksson

**Affiliations:** ^1^ Department of Molecular Medicine and Surgery Section of Sports Medicine Karolinska Institutet Stockholm Sweden; ^2^ Capio Artro Clinic and Stockholm Sports Trauma Research Center (SSTRC) FIFA Medical Centre of Excellence Sophiahemmet Stockholm Sweden; ^3^ Department of Orthopaedics Stockholm South Hospital Karolinska Institutet Stockholm Sweden

## Abstract

The menisci play a critical role in load transmission and shock absorption and serve as important secondary stabilizers of the knee. Unfortunately, meniscal repair is not always feasible, and resection may be required. Meniscal allograft transplantation is an established method of meniscal substitution; however, its use remains limited by graft sizing challenges, high costs, and restricted availability. The semitendinosus tendon has the capacity to remodel and revascularize within the intra‐articular environment. The purpose of this technical note is to describe the surgical technique for meniscal substitution using a semitendinosus tendon autograft.

## Graphical abstract

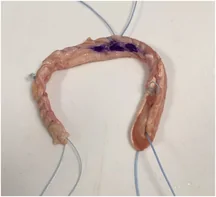

**VIDEO 1:** This is a technique video describing meniscal substitution with a semitendinosus tendon autograft. Shown here are the authors’ disclosures. The semitendinosus tendon graft is harvested with a tendon stripper in the same manner as for anterior cruciate ligament reconstruction. Care is taken to carefully remove any residual muscle tissue. The graft is folded to form a double‐stranded construct, and a No. 2 suture tape is passed through the loop. The graft is secured with a running 2‐0 FiberWire, and a No. 2 suture tape is applied to create a Chinese finger‐trap configuration on the free ends of the graft. The graft diameter is measured in the same manner as for anterior cruciate ligament reconstruction; in this case, it measured 6.5 mm on both ends. Anteromedial and anterolateral peripatellar portal are made, and any remaining native meniscal tissue is debrided with a motorized shaver to expose fresh tissue. Medial pie‐crusting can be performed to improve access, and anatomical posterior meniscal root tunnel is created. Anatomical anterior meniscal root tunnel is then created using a retrograde technique with a flip‐cutter; however, a full‐length tunnel drilling technique can also be used (which is optional). The graft is then introduced into the joint through the anteromedial portal, with the looped end guided into the posterior root tunnel. The graft is carefully seated against the meniscocapsular junction with the aid of a blunt instrument. The opposite end of the graft is pulled into the anterior root tunnel, and the graft is secured to the meniscocapsular junction with a combination of all‐inside and outside‐in vertical “hay bale” sutures encompassing the entire graft circumference. The meniscal root sutures are then maximally tensioned and fixed over a Tightrope attachable button system over the anterior aspect of the tibia. Final arthroscopic inspection confirms satisfactory graft position and appearance. Second‐look arthroscopy at 8 months postoperatively shows complete graft integration to the meniscocapsular junction and stability of the graft construct. Video content can be viewed at https://doi.org/10.1002/atn2.70088.

Loss of meniscal tissue has been associated with pain, impaired knee function, and a substantially increased risk of developing osteoarthritis.^1^, ^2^ In addition, the menisci serve as important secondary stabilizers of the knee.^3^, ^4^, ^5^ The primary objectives of meniscal substitution procedures are to restore, at least in part, the lost meniscal functions, alleviate patient symptoms, and provide a potential chondroprotective benefit.^6^, ^7^ Meniscal allograft transplantation is an established technique for meniscal substitution, with several studies showing favorable clinical outcomes.^8^, ^9^ Nevertheless, this technique is limited by regulatory restrictions in certain countries, technical complexity, graft sizing challenges, high costs, limited availability, and the potential risk of disease transmission.^10^ Studies in animal models have shown that tendon autografts can remodel into meniscus‐like structures, exhibiting composition and biomechanical properties comparable to those of the native meniscus.^11^, ^12^ This technical note outlines the surgical technique for meniscal substitution using a semitendinosus tendon (ST) autograft.

## SURGICAL TECHNIQUE

An illustration of the surgical technique is provided in Video 1. Key technical pearls and potential pitfalls are summarized in Table 1.

**TABLE 1:** Technical Pearls and Pitfalls of Meniscal Substitution Using a Semitendinosus Tendon Autograft

Pearls	Pitfalls
Meticulous graft preparation	Suboptimal graft preparation may lead to poor adaptation at the meniscocapsular junction or uneven tension distribution across the graft
Correct meniscal root tunnel placement	Incorrect placement of the meniscal root tunnels may compromise restoration of load distribution and shock absorption in the tibiofemoral compartment
Excise remaining native meniscal tissue and debride the meniscocapsular junction to expose fresh tissue	Incomplete removal of native meniscal tissue or inadequate debridement of the meniscocapsular junction may compromise graft healing
Use a combination of inside‐out, all‐inside, and outside‐in vertical “hay bale” sutures along the entire graft circumference to secure the graft to the meniscocapsular junction	Using a meniscal suture technique that is not tailored to the specific location (posterior horn, body, or anterior horn) may lead to suboptimal fixation, compromising graft stability and healing
Maximally tension both the anterior and posterior root sutures	Insufficient or uneven tensioning of the meniscal roots can prevent restoration of hoop stresses and compromise the effective distribution of axial loads across the tibiofemoral joint

### Patient Preparation

The patient is placed in the supine position on the operating table. A tourniquet is applied but usually not inflated, as the procedure is commonly performed without its use. To maintain 90° of knee flexion during graft harvesting, both a foot support and a lateral thigh support are positioned. The limb is then prepared and draped using standard sterile technique. Antibiotic prophylaxis is administered intravenously with 2 g of cloxacillin.

A comprehensive clinical and arthroscopic evaluation is performed, and any associated ligamentous injuries, cartilage lesions, or knee malalignment is addressed as appropriate.

### Graft Harvesting and Preparation

**FIGURE 1:**
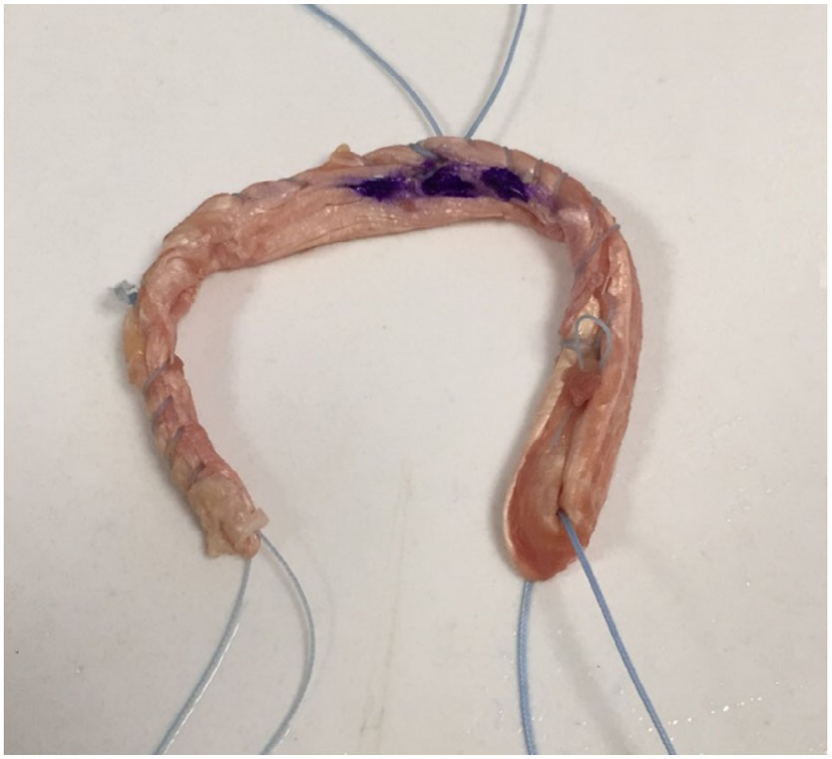
Prepared semitendinosus tendon autograft (Reproduced from Rönnblad E, Rotzius P, Eriksson K. Autologous semitendinosus tendon graft could function as a meniscal transplant. Knee Surg Sports Traumatol Arthrosc. 2022;30:1520‐1526).

The ST graft is harvested with a tendon stripper in the same manner as for anterior cruciate ligament reconstruction. After removal of residual muscle tissue, the graft is folded to form a double‐stranded loop and secured with a running 2‐0 FiberWire suture (Arthrex, Naples, FL, USA). A No. 2 suture tape is then applied to create a Chinese finger‐trap configuration on the free ends of the graft, while an additional FiberWire suture is placed through the loop (Figure 1). Nonresorbable FiberWire sutures can be added to each graft end to provide supplementary fixation strength.

### Arthroscopic Preparation, Tunnel Drilling, and Graft Placement

**FIGURE 2:**
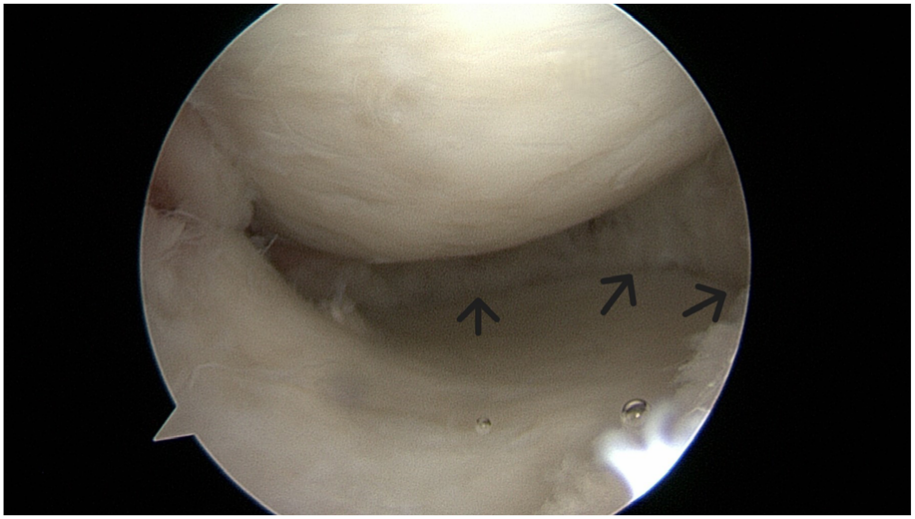
Right knee arthroscopic view of the medial meniscus from the anterolateral portal showing excision of the native meniscal tissue and debridement of the meniscocapsular junction to expose fresh tissue (black arrows).

Standard anteromedial and anterolateral arthroscopic portals are established. Any remaining native meniscal tissue is excised, and the meniscocapsular junction is debrided to expose fresh, bleeding tissue (Figure 2). If visualization of the medial compartment is restricted, medial pie‐crusting is performed to improve access.

**FIGURE 3:**
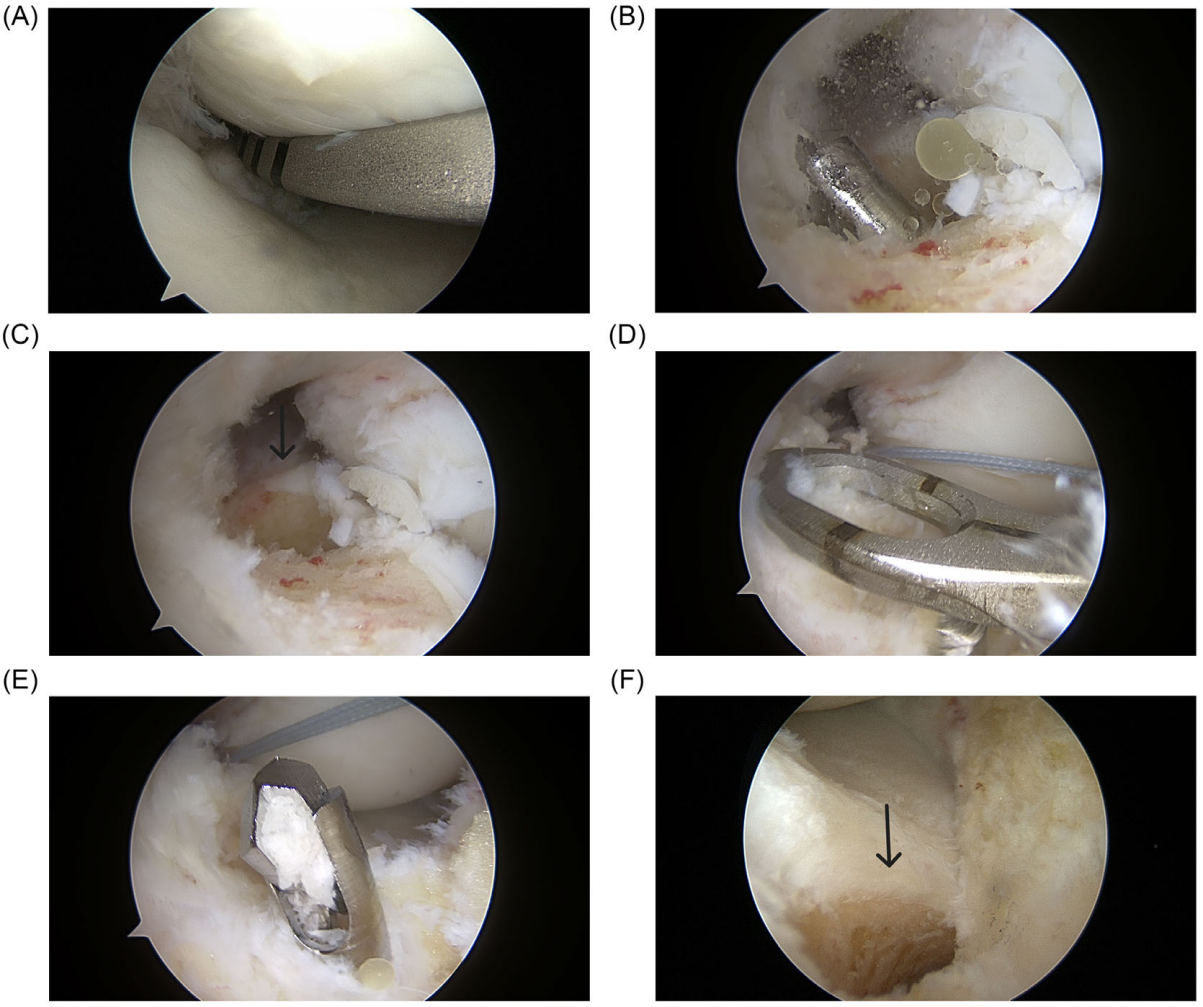
Right knee arthroscopic view of the medial compartment showing positioning of the meniscal root guide at the anatomic attachment of the posterior medial meniscal root (A), followed by tunnel drilling (B). Final appearance of the posterior meniscal root tunnel after anterograde drilling, matching the graft diameter (black arrow) (C). Positioning of the ACL tibial guide at the anatomic attachment of the anterior medial meniscal root (D), followed by retrograde tunnel drilling using a flip cutter matching the graft diameter (E). Final appearance of the anterior meniscal root tunnel (black arrow) (F). (ACL, anterior cruciate ligament.)

Anatomical meniscal root tunnels are created with either a meniscal root or anterior cruciate ligament tibial guide (Figure 3A‐F) and drilled to match the graft diameter, using either a retrograde technique with a flip‐cutter (Arthrex, Naples, FL, USA) or an anterograde approach with full‐length tunnels.

**FIGURE 4:**
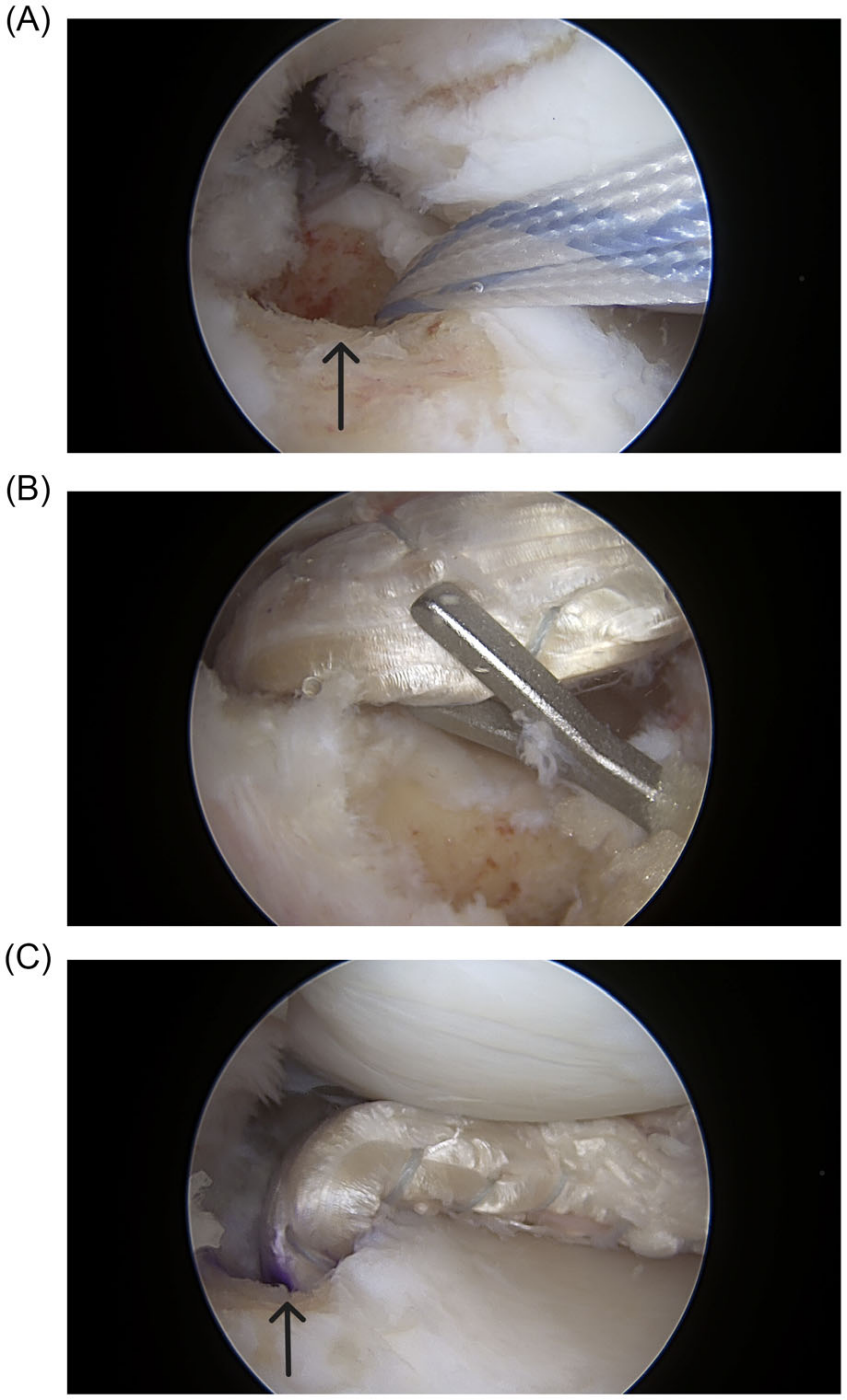
Right knee arthroscopic view of the medial compartment showing introduction of the graft into the joint through the anteromedial portal and guidance of the looped end into the posterior root tunnel (black arrows) under arthroscopic visualization (A‐C).

**FIGURE 5:**
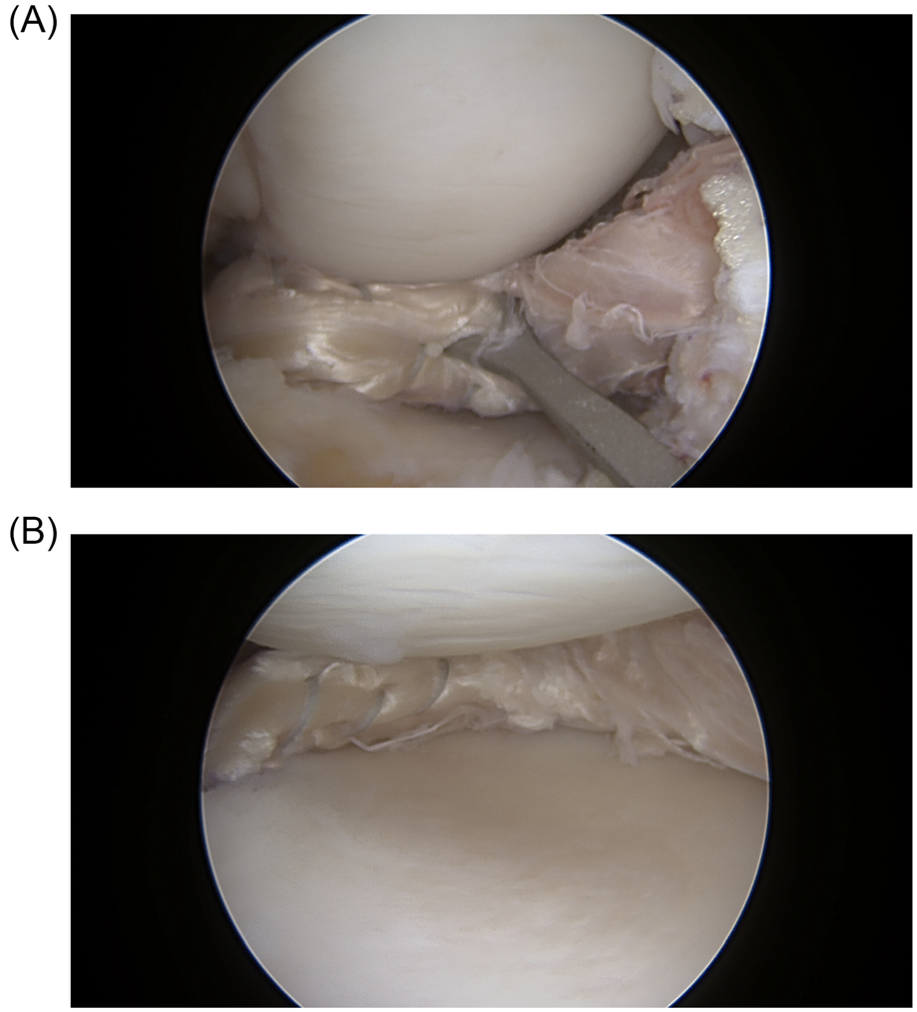
Right knee arthroscopic view of the medial compartment showing careful seating of the graft against the meniscocapsular junction with the assistance of a blunt arthroscopic instrument to ensure proper adaptation (A,B).

**FIGURE 6:**
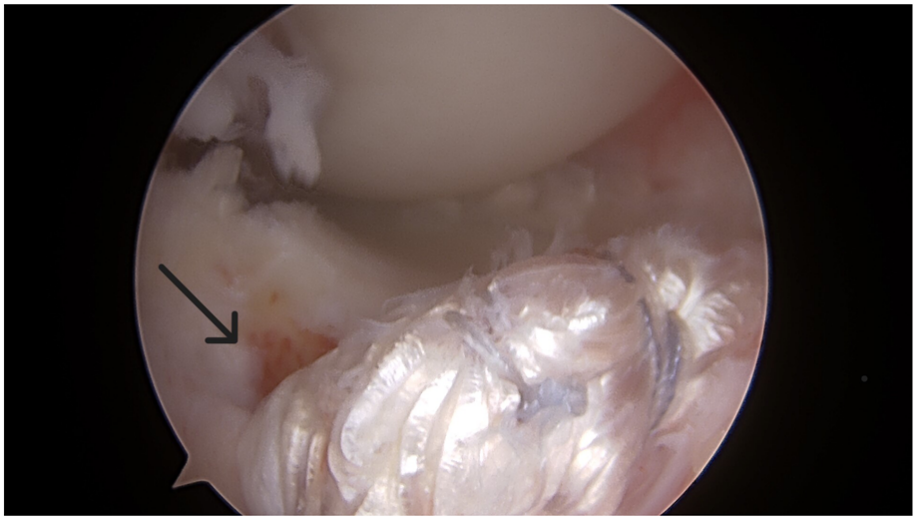
Right knee arthroscopic view of the medial compartment showing the remaining free end of the graft being pulled and seated within the anatomically prepared anterior meniscal root tunnel (black arrow).

**FIGURE 7:**
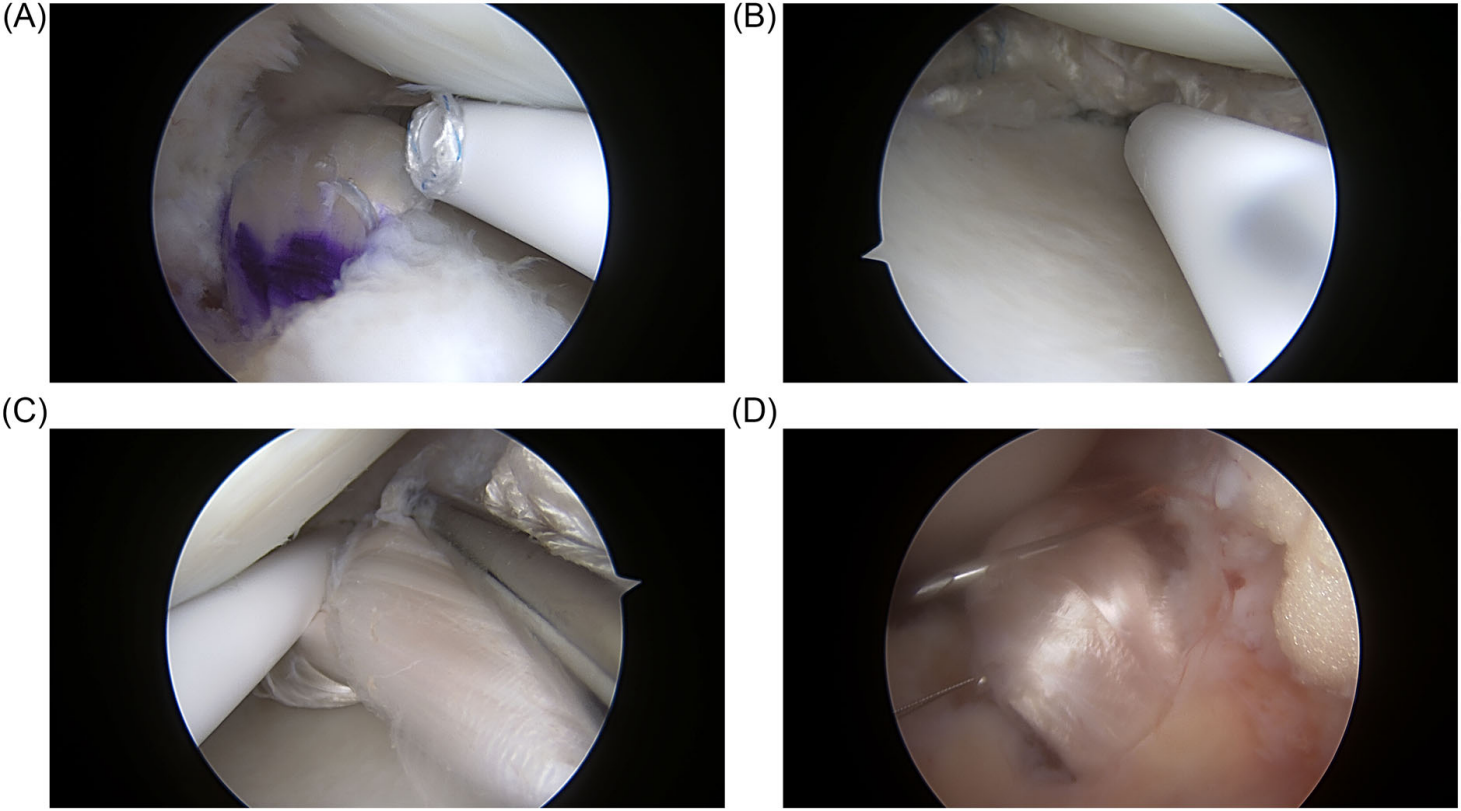
Right knee arthroscopic view of the medial compartment showing fixation of the graft to the meniscocapsular junction using a combination of all‐inside and outside‐in vertical “hay bale” sutures, ensuring circumferential stabilization of the entire graft (A‐D).

**FIGURE 8:**
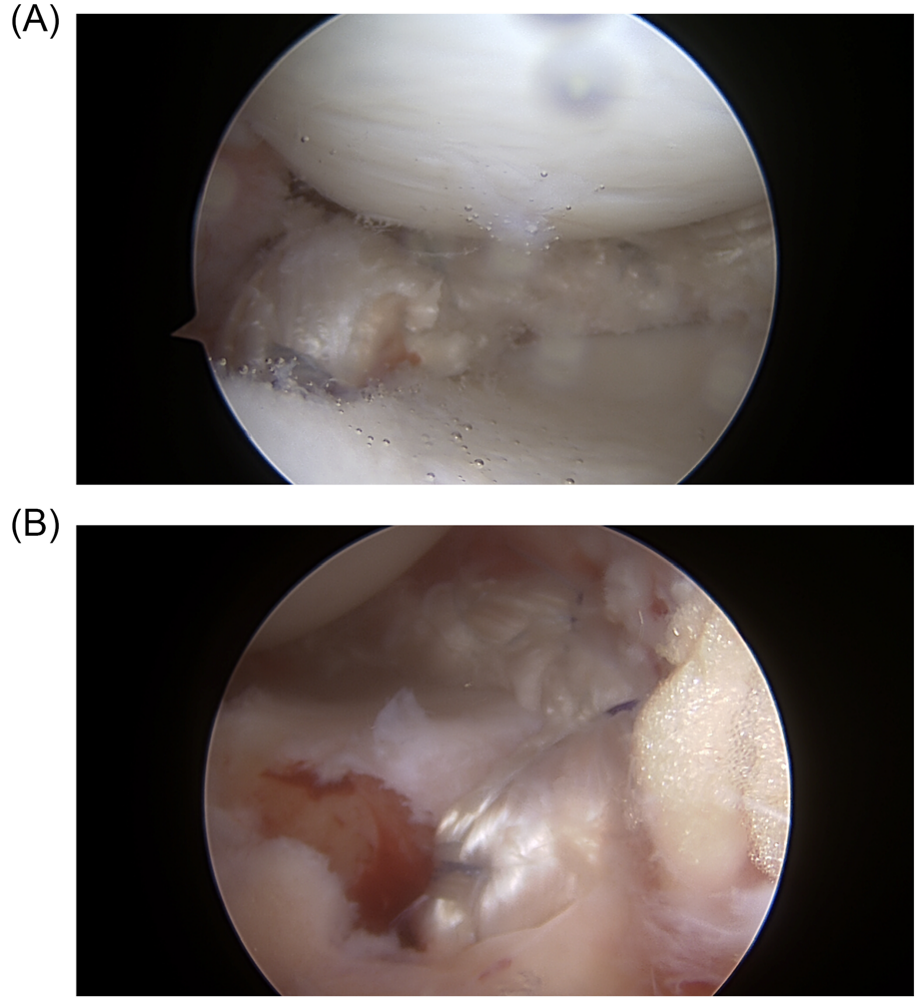
Right knee arthroscopic view of the medial compartment showing the final appearance of the graft seated against the meniscocapsular junction, with suture placement in the posterior horn (A) and in the midbody and anterior horn (B).

The graft is introduced into the joint via either the anteromedial or anterolateral portal. The looped end is guided into the posterior root tunnel (Figure 4A,B), and the graft is seated against the meniscocapsular junction with the aid of blunt instruments (Figure 5A,B). Finally, the opposite end of the graft is pulled into the anterior root tunnel (Figure 6). The graft is then secured to the meniscocapsular junction using a combination of inside‐out, outside‐in, and all‐inside vertical “hay bale” sutures, encompassing the entire graft circumference (Figure 7A‐D). Both meniscal roots are initially secured with temporary fixation. The sutures are then sequentially tensioned against the meniscal rim and capsule, after which the posterior and anterior root sutures are maximally tensioned and fixed over a Tightrope attachable button system (Arthrex, Naples, FL, USA) over the anterior aspect of the tibia. The final appearance of the graft is shown in Figure 8A,B.

### Postoperative Rehabilitation

Partial weight‐bearing is advised for the first 6 weeks following surgery. A hinged knee brace is worn for a total of 10 weeks, with progressive range‐of‐motion restrictions: 0° to 30° during weeks 1 to 2, 0° to 60° during weeks 3 to 4, and 0° to 90° during weeks 5 to 6. For the remaining 4 weeks, the brace is left unlocked to allow full range of motion. Deep squatting beyond 90° of knee flexion should be avoided for the first 4 postoperative months.

## DISCUSSION

Loss of meniscal tissue may lead to knee pain and elevate the risk of developing osteoarthritis.^1^ Meniscal substitution is an established option for patients experiencing ongoing medial or lateral tibiofemoral pain following subtotal or total meniscectomy. The primary objectives of this procedure are to alleviate symptoms, restore knee joint contact mechanics and laxity, and safeguard the articular cartilage from progressive damage. Meniscal allograft transplantation has been reported with favorable outcomes in numerous studies.^6^, ^9^, ^13^ Nevertheless, the procedure is associated with several challenges, including technical complexity, regulatory restrictions in certain regions, graft sizing and sterilization concerns, risk of disease transmission, and considerable costs.^10^ Furthermore, in many countries, access to suitable allografts remains limited. Meniscal substitution using an ST autograft offers several advantages. The graft is immediately available and eliminates concerns related to sterilization or disease transmission. In addition, sizing is not problematic, as the ST is versatile and can be tailored to fit knees of varying dimensions. Finally, the technique is considerably more cost‐effective than meniscal allograft transplantation. In a rabbit model, Li et al.^12^ revealed that ST autografts shared similar histological characteristics and biomechanical properties with the native meniscus. A recent cadaveric study^14^ reported that lateral meniscal substitution using a doubled ST autograft after total lateral meniscectomy markedly enhanced joint kinematics and nearly restored tibiofemoral contact mechanics to native conditions. A recent technical report^15^ on 4 patients with 12‐month follow‐up revealed that the transplant remained viable and adapted its form and function to resemble the native meniscus. Moreover, no adverse events were observed, and patients experienced improvements in both pain and quality of life. The advantages of this technique include ready graft availability, absence of sterilization and sizing issues, adaptability to knees of varying dimensions, no risk of disease transmission, and low costs. Potential disadvantages include the absence of true meniscal tissue, technical complexity, the risk of improper graft tensioning, donor‐site morbidity, and the lack of long‐term clinical outcome data (Table 2).

**TABLE 2:** Advantages and Disadvantages of Meniscal Substitution Using a Semitendinosus Tendon Autograft

Advantages	Disadvantages
Graft readily available	Lack of true meniscal tissue
No sterilization issues	Technically challenging
No sizing issues	Risk of improper graft tensioning
Graft can be adapted to fit knees with varying dimensions	Donor‐site morbidity
No potential disease transmission	No long‐term clinical data available
Low costs	

## DISCLOSURES

The authors (R.C., K.E.) declare the following financial interests/personal relationships which may be considered potential competing interests: R.C. reports a relationship with ESSKA Basic Science Committee, *KSSTA* Editorial Board, ISAKOS Young Professional Task Force Committee that includes: leadership or fiduciary role. K.E. reports a relationship with Arthrex, Episurf, and Personalized Knee that includes: consulting fees; Board of Swedish ACL Registry that includes: participation on a Data Safety Monitoring Board or Advisory Board; General Secretary of ESSKA and *KSSTA* Editorial Board that includes leadership or fiduciary role.
